# Conversion and reversion of anti‐John Cunningham virus antibody serostatus: A prospective study

**DOI:** 10.1002/brb3.1332

**Published:** 2019-06-06

**Authors:** Michael Auer, Harald Hegen, Johann Sellner, Katrin Oppermann, Gabriel Bsteh, Franziska Di Pauli, Thomas Berger, Florian Deisenhammer

**Affiliations:** ^1^ Department of Neurology Medical University of Innsbruck Innsbruck Austria; ^2^ Department of Neurology Christian Doppler Medical Center, Paracelsus Medical University Salzburg Austria

**Keywords:** antibodies, JCV, multiple sclerosis, natalizumab, progressive multifocal leukoencephalopathy, risk

## Abstract

**Introduction:**

Determination of antibodies against the John Cunningham virus (JCV) is an important tool for risk stratification in Natalizumab‐treated multiple sclerosis (MS) patients. Six‐monthly testing has been suggested for anti‐JCV antibody negative patients and patients with low antibody index in order to detect changes of serostatus. We conducted a prospective study with predefined testing intervals in order to investigate the predictability of anti‐JCV antibody status and the intervals for repetitive testing.

**Methods:**

Our study included 109 patients at the MS Clinic of the Departments of Neurology, Medical Universities of Innsbruck and Salzburg. Blood withdrawals were performed at five time points: baseline, month 1, 3, 6, and 12. Patients’ sera were sent to Unilabs, Copenhagen, Denmark, where anti‐JCV antibodies were tested by a two‐step enzyme‐linked immunosorbent assay. Qualitative (negative/positive) and quantitative results (anti‐JCV antibody index) were used for statistical analyses.

**Results:**

In our cohort, 52.3% of the patients were positive for anti‐JCV antibodies at baseline, with a significant correlation with age, but no association with sex or prior disease‐modifying therapy. Seven patients converted and reverted from negative to positive status and vice versa around the cut‐off index of 0.4, but no patient showed a permanent seroconversion from negative to highly positive anti‐JCV antibody status.

**Conclusion:**

Long‐term anti‐JCV antibody status, including seroconverters/‐reverters around the cut‐off index, is highly predictable by testing three times within short intervals, however, we cannot suggest clearly defined intervals for repetitive testing. The rate of real seroconverters, i.e., new infections with JCV, per year seems lower than previously described.

## 1 INTRODUCTION

John Cunningham virus (JCV) is known to cause the rare, but potentially life‐threatening progressive multifocal leukoencephalopathy (PML) in Natalizumab‐treated multiple sclerosis (MS) patients (Bloomgren et al., 2012). Therefore, detection of anti‐JCV antibodies in patients’ serum is an important tool for risk stratification and, consequently, treatment decisions in clinical routine. A standardized two‐step enzyme‐linked immunosorbent assay (ELISA) is preferably used (Lee et al., 2013). Test results are reported qualitatively (negative or positive) and semiquantitatively as anti‐JCV antibody index in antibody positive patients only, the latter expressed by the optical density (OD) value of the ELISA, which can yield values between 0.2 and approximately 6.0. In general, index values below 0.2 are considered negative, over 0.4, positive. For indices between 0.2 and 0.4, a confirmation assay is performed and, according to that result, the serostatus can be determined as negative or positive for values within this range. In previous studies, around 55%–60% of MS patients have been tested positive for anti‐JCV antibodies (Bozic et al., 2014; Olsson et al., 2013; Outteryck et al., 2012; Warnke et al., 2012). Additionally, it turned out that the anti‐JCV antibody index can be used for PML risk stratification (Plavina et al., 2014). Most patients who eventually developed PML showed an index value of 1.5 or higher, thus anti‐JCV antibody positive patients can be stratified in a low and high PML‐risk group by anti‐JCV antibody index (Ho et al., 2017). Furthermore, longitudinal observations of multiple JCV testing showed that most patients present stable anti‐JCV antibody status and index values over years, although seroconvertion rates of 5%–33% per year have been described (reviewed by Schwab et al., 2017). In this context one has to distinguish between patients who fluctuate around the cut‐off index of the ELISA test, by that converting and reverting between anti‐JCV antibody negative and positive although showing index values within a narrow range, and patients who experience a new infection with JCV by converting from negative to persistently positive anti‐JCV antibody index values (Alroughani et al., 2016; Campagnolo, Ho, & Patel, 2016; Donovan & LaGanke, 2016; Hegen et al., 2017; Plavina et al., 2014; Vennegoor et al., 2016). So far, in Natalizumab‐treated MS patients who are negative or low positive for anti‐JCV antibodies, six‐monthly follow‐up tests are recommended by consensus (McGuigan et al., 2016), in order to detect seroconverters and to discuss treatment continuation in patients with increased risk of PML. However, there is no scientific evidence for six‐monthly test intervals as these time intervals have not been investigated in longitudinal studies so far. We conducted the first prospective study which included MS patients on different disease‐modifying therapies and performed follow‐up testing of anti‐JCV antibodies in predefined time intervals.

As a primary goal we wanted to investigate if changes of serostatus between negative, positive, and vice versa at different levels of the anti‐JCV antibody index over 12 months could be also seen in short‐term intervals which would support an assay related variation rather than a true conversion.

Additionally, we wanted to confirm development of anti‐JCV antibody index values over time in a prospective setting, which has been well described in retrospective analyses (Hegen et al., 2017; Schwab et al., 2017).

## 2 METHODS

### 2.1 Study population

This prospective study was conducted at the MS Clinic of the Departments of Neurology, Medical University of Innsbruck and Medical University of Salzburg. Between December 2014 and January 2016, 110 patients were included, according to a power calculation based on the so far published JCV prevalence and seroconversion data. Inclusion criteria were the diagnosis of clinically definite relapsing‐remitting MS according to the McDonald criteria 2010 (Polman et al., 2011) and the willingness of giving blood samples at the defined time points. Patients were allowed to be treated with any disease‐modifying drug (DMD) except for intravenous immunoglobulins as this was described to potentially influence the anti‐JCV antibody serostatus (Kister et al., 2014). Moreover, no patients treated with immunosuppressive drugs such as Cyclophosphamide, Mitoxantrone, and Rituximab were included into the study. The study was approved by the ethics committee of the Medical University of Innsbruck; all patients gave written informed consent before being enrolled into the study.

### 2.2 Sampling

A blood withdrawal was performed at five predefined time points: At baseline visit (month 0), after 4–8 weeks (month 1) and at months 3, 6, and 12. We used the sampling kits provided from Unilabs, Copenhagen, and sent the samples to the laboratory the same day of withdrawal, so that laboratory analyses could be started within 48 hr after sampling. Anti‐JCV antibodies were measured, by a two‐step ELISA (Lee et al., 2013). Qualitative (negative/positive) and, for anti‐JCV antibody positive patients, semi‐quantitative results (i.e., anti‐JCV antibody index which is the OD value of the ELISA) were obtained.

### 2.3 Statistics

For statistical analysis Graph Pad Prism 6 (Graphpad Software Inc, La Jolla, CA, USA) was used. Distribution of data was tested using D'Agostino‐Pearson normality test. According to distribution, data are shown either as median and range or mean ± standard deviation as appropriate. Association between sex and anti‐JCV antibody status was analyzed using Chi‐square test, age and anti‐JCV antibody status with paired *t* test. Correlation of age and JCV index was analyzed with Spearmen correlation test. Mann‐Whitney‐test was used for comparison of anti‐JCV antibody negative and positive patients regarding high‐dose intravenous methylprednisolone (HDMP) therapy before study and Fisher's exact test for association between HDMP during study and serostatus change. Two‐tailed *p*‐values of <0.05 were considered statistically significant.

## 3 RESULTS

One hundred and ten patients were included in the study, 93 at the Medical University of Innsbruck and 17 at the Medical University of Salzburg. One patient was lost to follow‐up, so that a total of 109 patients finished the study per protocol. Of these, 52 (47.7%; 95%‐CI: 38.9%–57.5%) were anti‐JCV antibody negative and 57 (52.3%, 95%‐CI: 42.5%–61.9%) positive at the baseline visit. While 51 patients (46.8%) remained consistently anti‐JCV antibody negative and 51 (46.8%) consistently positive throughout the study, seven of 109 patients (6.4%, 95%‐CI: 2.6%–12.8%) changed serostatus during the study, i.e., converted and reverted between negative and positive serostatus. No patient in our cohort converted from negative to positive anti‐JCV antibody status and remained consistently positive thereafter. The prevalence of anti‐JCV antibodies and number of patients switching between negative and positive serostatus or fluctuating below and above a particular anti‐JCV antibody index, respectively, are shown in Table 1. The seven patients with changing anti‐JCV antibody serostatus were on different disease‐modifying treatments during the study: four on Natalizumab, one on Dimethyl fumarate, one changed from Glatirameracetate to Dimethyl fumarate during the study and one had no treatment at baseline but initiated Dimethyl fumarate during the study. Table 2 shows development of anti‐JCV antibody serostatus and index of these seven patients at all single visits. The maximum anti‐JCV antibody index in all these seven patients was 0.53. Analogously, in Table 3 we present the index values of those six patients who con‐/reverted around an assumed cut‐off index of 0.9 and 1.5, respectively, representing different PML‐risk groups.

**Table 1:** JCV prevalence data and consistency of anti‐JCV antibody index throughout the study for different cut‐off indices

Cut‐off index	Below cut‐off	Above cut‐off	"Switcher"
0.4	51 (46.79)	51 (46.79)	7 (6.42)
0.9	66 (60.55)	41 (37.61)	2 (1.83)
1.5	68 (62.39)	37 (33.94)	4 (3.67)

Note

The table shows the number (percentage) of patients who stayed consistently below or above the indicated anti‐JCV antibody index cut‐off. “Switcher” are those patients who converted or reverted between anti‐JCV antibody‐negative and ‐positive serostatus throughout the study without remaining consistently positive after seroconversion. For the cut‐off of 0.4 these are the permanently anti‐JCV antibody negative and positive patients. For the cut‐off‐values of 0.9 and 1.5, which may distinguish between positive with low PML‐risk and positive with high PML‐risk, the patients were divided the same way into below and above the cut‐off index or switching around the indicated cut‐off.

Abbreviations: JCV, John Cunningham virus; PML, progressive multifocal leukoencephalopathy.

**Table 2:** Anti‐JCV antibody test results at single visits of the seven patients who con‐/reverted between negative and positive anti‐JCV antibody status

Month	0	1	3	6	12
Patient 1	0.53	Neg	0.41	Neg	Neg
Patient 2	0.27	Neg	Neg	Neg	Neg
Patient 3	0.46	0.45	Neg	0.45	0.48
Patient 4	0.42	Neg	Neg	Neg	Neg
Patient 5	Neg	Neg	0.51	Neg	Neg
Patient 6	0.32	Neg	Neg	Neg	Neg
Patient 7	0.51	0.47	Neg	Neg	0.43

Note

Negative test results are not further specified by an exact index value, whereas index values for positive test results are shown. Cut‐off index of 0.2–0.4 is predefined by the Stratify ELISA (Lee et al., 2013).

Abbreviations: ELISA, enzyme‐linked immunosorbent assay; JCV, John Cunningham virus.

**Table 3:** Anti‐JCV antibody test results at single visits of the six patients who fluctuated around the cut‐off indices of 0.9 and 1.5, assuming different PML‐risk groups

Month	0	1	3	6	12
Patient 1	0.67	*1.09*	*1.02*	*1.29*	*1.07*
Patient 2	*1.21*	*1.29*	0.86	*0.94*	*1.34*
Patient 3	*1.51*	1.50	1.30	1.42	1.34
Patient 4	*1.59*	*1.52*	*1.96*	*1.78*	1.37
Patient 5	*1.75*	*1.53*	*1.92*	1.43	1.21
Patient 6	*1.57*	1.49	*1.70*	*1.56*	d.m.

Note

Patient 1 and 2 con‐/reverted around an index‐value of 0.9, the patients 3–6 around 1.5. For better visibility, index values above the cut‐off (0.9 and 1.5 respectively) are written in italic.

Abbreviations: d.m., data missing; JCV, John Cunningham virus; PML, progressive multifocal leukoencephalopathy.

In our study cohort 29 patients (26.6%) were male and 80 (73.4%), female. Of the 29 male patients, 11 (37.9%) were anti‐JCV antibody negative and 18 (62.1%) positive at baseline, one of the positive patients switched between negative and positive during the study. Of the 80 female patients, 41 (51.2%) were anti‐JCV antibody negative and 39 (48.8%) positive at baseline visit; during the study six of the female patients converted or reverted between negative and positive serostatus. There was no significant difference (*p* = 0.219) regarding gender and anti‐JCV antibody status or rate of serostatus change.

The mean age of our patients at baseline visit was 36.4 ± 8.5 years. Analyzing anti‐JCV antibody negative and positive patients separately, we found a mean age of 34.4 ± 8.7 years in the negative and 38.4 ± 8.2 in the positive group (*p* = 0.019). The seven converting/reverting patients had a mean age of 35.9 ± 6.0 years. There was a statistically significant correlation (*p* = 0.029, *r* = 0.211) between age and anti‐JCV antibody index at baseline visit.

The type of DMD at baseline visit and change of treatment during the study did not show any association with development of anti‐JCV antibody serostatus during the study. Table 4 displays DMD at baseline visit. During the study 22 of 109 patients (20.2%) changed the DMD, however, proportions of anti‐JCV antibody negative, positive and converting/reverting patients were similar as in the 87 patients who did not switch therapy. Of 49 patients treated with Natalizumab 32 (65.3%) were negative for anti‐JCV antibodies, whereas only two of 21 patients (9.5%) treated with Fingolimod were negative at baseline. For the other DMDs there was no difference between negative and positive patients.

**Table 4:** Disease‐modifying drugs (DMD) at baseline visit

DMD at baseline	*n*	%
Interferon‐beta	14	12.84
IFNβ‐1a 30 µg IM	8	
IFNβ‐1a 44 µg SC	3	
IFNβ‐1b 250 µg SC	2	
PegIFNβ‐1a 125 µg SC	1	
Glatirameracetate	11	10.09
Teriflunomide	2	1.83
Dimethylfumarate	7	6.42
Natalizumab	49	44.95
Fingolimod	21	19.27
No DMD	5	4.59

Overall median time from last HDMP course before study entry to baseline visit was 1.2 (0–15.2) years, 1.1 (0–10) in anti‐JCV antibody negative, and 1.2 (0–15.2) in positive patients (*p* = 0.979). During the study 26 of 109 (23.9%) patients received standard high dose methylprednisolone (HDMP) therapy for relapses. Of the seven seroconverters/reverters, three received HDMP and four patients did not (*p* = 0.364).

## 4 DISCUSSION

We investigated the longitudinal development of anti‐JCV antibody status and index in MS patients using predefined short‐termed intervals. This provides the advantage of clearly defined follow‐up periods of JCV testing in all patients, in contrast to recently published data on cohorts, where test intervals had to be defined retrospectively by approximation (Alroughani et al., 2016; Donovan & LaGanke, 2016; Hegen et al., 2017; Plavina et al., 2014; Vennegoor et al., 2016). Regarding demographic data, our study represents a typical MS population with a mean age in the midthirties and a predominance of female patients (Pugliatti et al., 2006). Regardless of DMD, most subjects showed stable anti‐JCV antibody status over time. We did not find any sign of a higher seroconversion rate in Natalizumab‐treated patients as it was described in previous studies (Hegen et al., 2017; Schwab et al., 2016; Vennegoor et al., 2016; Warnke et al., 2013), with the limitation that our study was not powered for treatment questions. We observed different anti‐JCV antibody prevalence at baseline between the Natalizumab and Fingolimod group. While in the Natalizumab group the prevalence of JCV was lower than in the general study population, almost all (19 of 21) patients in the Fingolimod group were anti‐JCV antibody positive. This reflects the treatment decisions based on anti‐JCV antibody status before including patients into this noninterventional study, leading to a selection bias regarding the overall prevalence of anti‐JCV antibodies in our study cohort. Due to the high number of Natalizumab‐treated patients in our cohort—because patients treated with Natalizumab were best accessible for short‐term blood withdrawal receiving monthly infusions—prevalence of JCV was approximately 52%, slightly lower than previously published data would show (Bozic et al., 2014; Olsson et al., 2013; Outteryck et al., 2012; Warnke et al., 2012). Anti‐JCV antibody status and index showed a significant correlation with age, as it was found in other cohorts as well (Hegen et al., 2017; Schwab et al., 2017).

In our cohort, all 109 patients showed stable anti‐JCV antibody index values during the study period of 12 months. The great majority of anti‐JCV antibody negative patients at baseline (all but one) remained negative at all time points of testing, while, similarly, all highly positive patients remained on high index throughout the study at all time points. There was no patient switching from negative to continuously high positive status, i.e., reflecting a new infection with JCV. This observation suggests that the rate of true seroconverters per year may be lower than discussed in previous publications (Schwab et al., 2017). We chose a study cohort of approximately 110 patients based on a power calculation assuming a seroconversion rate of 3% per year, as previously described (Hegen et al., 2017). The fact that no patient switched from negative to permanently positive serostatus reflects the lowest expected conversion rate within a 95% confidence interval of 0.0%–6.8%. However, this finding underlines that the rate of real seroconverters, i.e., new infections with JCV during the observation time might be lower than estimated in other studies, whereas, a conversion rate of around 3% or even lower seems to be expected. All seroconverters and reverters that we observed, fluctuated around the cut‐off index of 0.2/0.4. In seven patients, we found this described phenomenon of switching between negative and positive antibody status while remaining within a stable range of JCV‐index around 0.4. In these seven patients, the highest index observed was 0.53, which is much below the threshold of 1.5 that has been suggested for discrimination between low and high PML‐risk patients (Ho et al., 2017; Plavina et al., 2014). Analogously, when using other thresholds, such as 0.9 and 1.5, representing different PML‐risk groups, we observed a few patients fluctuating around this index, remaining within a stable index range. Most of previously described rates of seroconverters were based on change of anti‐JCV antibody status only, without considering the index (reviewed by Schwab et al., 2017). Therefore, these rates may mostly reflect patients who switch around the cut‐off index without truly converting from negative to clearly positive antibody status and, thus, overestimating the rate of new JCV infections per year.

The main objective of this study was to investigate whether changes of serostatus between negative, positive and vice versa could be also seen in short‐term intervals. In those patients who changed serostatus during the study around the cut‐off, the conversion was seen at any time point, also in short‐term intervals, i.e., within one month in some cases, with fast reversion thereafter. This finding underlines the hypothesis, that most of serostatus changes are likely due to assay variation and do not reflect a true infection with JCV. Corresponding coefficients of variation (CV), such as intra assay CV of 3.2 or inter assay CV of 5.9% (details see Lee et al., 2013) emphasize this hypothesis.

Additionally, we were interested, whether it would be possible to predict long‐term anti‐JCV antibody status by testing patients three times within 3 months (baseline, 1 and 3 months) and comparing these results with six‐monthly testing (baseline, 6 and 12 months). In 102 of 109 patients, anti‐JCV antibody status was the same at any time point of testing during the study. The seven “switchers” would have been all identified during the first 3 months as well (see Tables 2 and 3), so that for all 109 patients JCV testing at months 6 and 12 did not add any additional benefit. The idea of testing three times in short intervals rose by considering the sensitivity and specificity data of the JCV‐ELISA (Berger & Fox, 2016; Lee et al., 2013; O'Connor & Kremenchutzky, 2015), which allows a reliability of the test results of more than 95% when the result is confirmed at three consecutive occasions. By testing for anti‐JCV antibodies three times in short intervals, serostatus fluctuation around the cut‐off due to assay variability and not due to a new infection can be identified already within the first months. The consensus of testing for anti‐JCV antibodies every 6 months (McGuigan et al., 2016, O'Connor & Kremenchutzky, 2015) negative (and low positive, i.e., low‐PML risk) patients, is not based on observations of long‐term serostatus development, since there has been no study investigating appropriate test intervals. Our study shows that there is no exact testing interval that could be recommended based on scientific evidence, since rate of real seroconverters seems very low. However, the six‐monthly testing can be supported by clinical observations that show that there have been very few PML cases in patients previously tested negative for JCV when using six‐monthly test intervals.

## 5 CONCLUSION

With this first prospective study regarding longitudinal follow‐up of anti‐JCV antibody status and index in predefined test intervals, we were able to show that a longer‐term serostatus is highly predictable by testing three times within short intervals—including identification of serostatus fluctuation due to assay variability without real seroconversion. Most of serostatus changes with stable index value levels originate from the test variability of the assay and not from new JCV infections. For patients with index values around the cut‐off, short testing intervals may be a tool for clinical routine, however, it does not replace the consecutive testing of anti‐JCV antibodies in negative and low positive patients in longer intervals for detection of possible and rare seroconversion due to a new infection with JCV.

## CONFLICTS OF INTEREST

The authors do not report any conflicts of interest regarding this study. Outside the study, the authors report following conflicts of interest. M. Auer has participated in meetings sponsored by, received speaker honoraria or travel funding Novartis, Biogen, and Merck Serono. H. Hegen has participated in meetings sponsored by, received speaker honoraria or travel funding from Bayer Schering, Biogen, Merck Serono, and Novartis, and received honoraria for acting as consultant for Teva Pharmaceuticals Europe. J. Sellner received speaker honoraria from Biogen, Merck, Sanofi, Novartis, Roche, Medday, Teva‐Ratiopharm, his institution received unrestricted grants from Biogen, Roche and Sanofi. K. Oppermann reports no disclosures. G. Bsteh has participated in meetings sponsored by, received speaker honoraria or travel funding from Biogen, Merck Serono, Novartis, Genzyme and Teva Ratiopharm, and received honoraria for acting as consultant for TevaPharmaceuticals Europe. F. Di Pauli has received speaking honoraria or travel funding from Biogen‐Idec, Roche Austria, and Sanofi‐Aventis Austria. T. Berger has participated in the last 2 years in meetings sponsored by and received honoraria (lectures, advisory boards, consultations) from pharmaceutical companies marketing treatments for multiple sclerosis: Almirall, Biogen, Bionorica, Celgene, MedDay, Merck, Novartis, Roche, Sanofi Aventis/Genzyme, TG Therapeutics, and TEVA. His institution has received financial support in the last 2 years by unrestricted research grants (Biogen, Novartis, Sanofi Aventis/Genzyme, Roche, TEVA) and for participation in clinical trials in multiple sclerosis sponsored by Alexion, Bayer, Biogen, Merck, Novartis, Roche, Sanofi Aventis/Genzyme, TEVA. F. Deisenhammer has participated in meetings sponsored by or received honoraria for acting as an advisor/speaker for Bayer Healthcare, Biogen Idec, Genzyme‐Sanofi, Merck, Novartis Pharma, and Teva‐Ratiopharm. His institution has received financial support for participation in randomized controlled trials of INFb‐1b (Betaferon, Bayer Schering Pharma), INFb‐1a (Avonex, Biogen Idec; Rebif, Merck Serono), glatiramer acetate (Copaxone, Teva Pharmaceuticals), Natalizumab (Tysabri, Biogen Idec), in multiple sclerosis. He is the section editor of the MSARD Journal (Multiple Sclerosis and Related Disorders).

## Data Availability

The data that support the findings of this study are available from the corresponding author upon reasonable request.
